# On the Difference Between Numerosity Processing and Number Processing

**DOI:** 10.3389/fpsyg.2018.01650

**Published:** 2018-09-12

**Authors:** Anne H. van Hoogmoed, Evelyn H. Kroesbergen

**Affiliations:** ^1^Department of Pedagogical and Educational Sciences, Utrecht University, Utrecht, Netherlands; ^2^Department of Special Needs Education and Youth Care, University of Groningen, Groningen, Netherlands; ^3^Behavioural Science Institute, Radboud University, Nijmegen, Netherlands

**Keywords:** number processing, ERP, ANS mapping account, non-symbolic, quantity processing, visual properties

## Abstract

The ANS theory on the processing of non-symbolic numerosities and the ANS mapping account on the processing of symbolic numbers have been the most popular theories on numerosity and number processing, respectively, in the last 20 years. Recently, both the ANS theory and the ANS mapping account have been questioned. In the current study, we examined two main assumptions of both the ANS theory and the ANS mapping account. ERPs were measured in 21 participants during four same-different match-to-sample tasks, involving non-symbolic stimuli, symbolic stimuli, or a combination of symbolic and non-symbolic stimuli (i.e., mapping tasks). We strictly controlled the visual features in the non-symbolic stimuli. Based on the ANS theory, one would expect an early distance effect for numerosity in the non-symbolic task. However, the results show no distance effect for numerosity. When analyzing the stimuli based on visual properties, an early distance effect for area subtended by the convex hull was found. This finding is in line with recent claims that the processing of non-symbolic stimuli may be dependent on the processing of visual properties instead of on numerosity (only). With regards to the processing of symbolic numbers, the ANS mapping account states that symbolic numbers are first mapped onto their non-symbolic representations before further processing, since the non-symbolic representation is at the basis of processing the symbolic number. If the non-symbolic format is the basic format of processing, one would expect that the processing of non-symbolic numerosities would not differ between purely non-symbolic tasks and mapping tasks, resulting in similar ERP waveforms for both tasks. Our results show that the processing of non-symbolic numerosities does differ between the tasks, indicating that processing of non-symbolic number is dependent on task format. This provides evidence against the ANS mapping account. Alternative theories for both the processing of non-symbolic numerosities and symbolic numbers are discussed.

## Introduction

A prominent view on number processing is that non-symbolic quantities are processed intuitively by the approximate number system (ANS; Dehaene, 1997). The numerosity of a set of objects is assumed to be approximated by this system. This ANS theory is confirmed in a number of studies in infants, showing sensitivity to the numerosity of a set of objects from 6 months of age (Xu and Spelke, 2000; Xu et al., 2005). Based on these studies, the processing of numerosity is assumed to be innate and shared across species (Xu and Spelke, 2000; Xu et al., 2005; Izard et al., 2009). Whereas the ANS theory concerns processing of the numerosity of sets of objects, an extension of the theory, named the ANS mapping account, is concerned with the processing of symbolic numbers. The ANS mapping account states that symbolic number processing is dependent on the ANS. Symbolic numbers that are encountered, are assumed to be first converted into a non-symbolic numerosity before further processing (Dehaene, 1997). Recently, both the ANS theory and the ANS mapping account have been questioned (Cohen Kadosh and Walsh, 2009; Gebuis et al., 2016; Lourenco et al., 2016; Reynvoet and Sasanguie, 2016; Leibovich et al., 2017; Núñez, 2017). The current study had two goals. First, we aimed to examine whether the processing of non-symbolic numerosity does indeed rely on an intuitive approximation of the numerosity of a set of objects, which would confirm the ANS theory. Second, we examined whether the processing of symbolic numbers is indeed based on the ANS as assumed by the ANS mapping account.

### The ANS Theory

The ANS theory has been the most influential account on numerosity processing for the last 20 years. It suggests that the numerosity of a set of objects is approximated by extracting the numerosity from this set of objects independently of the visual properties of the set. Based on a mental number line, numerosities can be compared to each other (Dehaene, 1997). The approximation means that a set of objects does not only activate the corresponding numerosity, but also numerosities that are nearby on the mental number line. As such a set of 15 objects does not only activate the quantity 15 on the mental number line, but also 14 and 16, and to a lesser degree, 13 and 17. This leads to overlapping neural representations of the numerosities 15 and 16, but not for example 15 and 30. The larger the numerosity to be estimated, the more neighboring numerosities are co-activated. This explains why it is harder to distinguish between 15 and 16 objects than between 15 and 30 objects, and harder to distinguish between 15 and 16 than between 5 and 6.

Evidence for the ANS theory is mainly based on the results of comparison tasks. In these tasks, two sets of dots are presented and participants have to decide which set contains the largest number of dots. Lower accuracy and longer reaction times are obtained when the ratio between two quantities is closer to 1. For example, it is more difficult to compare 6 vs. 8 dots (ratio 0.75) than to compare 4 vs. 8 dots (ratio 0.5), but also more difficult to compare 6 vs. 8 dots (ratio 0.75) than to compare 4 vs. 6 dots (ratio 0.66). This effect is called the ratio effect (Reynvoet and Sasanguie, 2016; Smets et al., 2016) and is thought to be due to the co-activation of numerosities that are close on the number line. The closer the numerosities are to each other, the more they co-activate the same numerosity, which makes it more difficult to decide which is the larger one, in turn resulting in lower accuracy and higher reaction times. This ratio effect is not limited to behavioral studies, but is also shown in ERP research, where the amplitudes of the ERP signal differ per ratio between two numerosities. More specifically, ERP studies on non-symbolic processing have shown ratio-dependent ERP amplitudes in varying time windows between 120 and 490 ms (Temple and Posner, 1998; Libertus et al., 2007; Paulsen and Neville, 2008; Hyde and Spelke, 2009, 2012). These ratio effects may reflect numerosity processing based on the ANS. However, the effects may also be due to the processing of the visual properties of the non-symbolic stimuli (i.e., a set of dots) instead of the numerosity of the sets.

In real life, visual properties of a set of objects co-vary with the number of objects in the set. For example, if you compare 5 fish to 10 fish, than the larger number of fish also occupies more of the visual scene, both in total surface of the fish as well as the area they occupy. Thus, in determining which group contains most fish, one could use both the visual properties (such as surface or area) as well as numerosity. The same holds for arrays of dots (or other non-symbolic stimuli). As such, it is difficult to distinguish the processing of visual input from the processing of numerosity. This problem has been acknowledged within the field for many years already (Mix et al., 2002). Different methods have been developed to control for visual input to be able to examine pure numerosity processing. Most ERP studies have used some sort of control for visual input when studying the processing of non-symbolic numerosities. An often-used method to control for effects of visual input has been described by Dehaene et al. (2005). Using this method, on half of the trials, the total surface of the dots or convex hull is equated, whereas the diameter of the dots and the distance between the dots varies. On the other half of the trials, diameter or distance between dots is equated, and total surface or convex hull varies. Studies using this type of control for visual input still show early ERP effects for small quantities (Libertus et al., 2007; Hyde and Spelke, 2012), which may suggest that numerosity processing is indeed automatic. However, these results may be due to the impossibility to strictly control for visual parameters when using small quantities. When using larger quantities, the early N1 effects disappeared, but distance effects were still found in the P2p time window, suggesting that numerosity is processed in a ratio-dependent manner in the latter time window (Libertus et al., 2007; Hyde and Spelke, 2012).

Gebuis and Reynvoet (2011) suggested that the control for visual input developed by Dehaene et al. (2005) may not be sufficient. Participants could not rely on a single visual property to compare numerosities, but could still use total surface or convex hull in half of the trials, and diameter or distance between the dots in the other half of the trials. Therefore, Gebuis and Reynvoet (2011) developed a more advanced method to control for visual properties in which all properties are varied simultaneously and visual properties only explain a very small portion of the variance in numerical distance (Gebuis and Reynvoet, 2011). When comparing this method with a method similar to the one developed by Dehaene et al. (2005), diverging results were found (Gebuis and Reynvoet, 2012). When using the method of Dehaene et al. (2005) N1 and P2 effects were found. When controlling for visual input with the method developed by Gebuis and Reynvoet (2011), no N1 and P2 effects were found, suggesting that the N1 and P2 effects found in the first experiment are explained by visual cues. Also other studies using this more stringent method of Gebuis and Reynvoet (2011) found distance effects only in later ERP components starting around 600 ms (Soltész and Szűcs, 2014), or no ERP components related to distance at all (Gebuis and Reynvoet, 2013). This suggests that the processing of non-symbolic stimuli is not based on the extraction of approximate numerosity, but instead relies on the processing of visual features.

Indeed the ANS has recently been questioned based on the abovementioned results (Gebuis et al., 2016; Leibovich et al., 2017; Núñez, 2017), and alternatives have been proposed. Gebuis et al. (2016) propose a sensory integration theory, in which visual properties are not removed in order to compare numerosity, but are a the basis of this comparison (see also Gevers et al., 2016). Different sensory cues are integrated to compare numerosities. Related to this theory, Leibovich et al. (2017) propose a sense for magnitude theory instead of a sense for number. This theory states that magnitude processing and not number processing is automatic and innate. They claim that the development of numerosity processing is based on this sense for magnitude as children discover the relation between numerosity and magnitude. However, several comments on this paper counter this idea by arguing that a sense of numerosity is innate and automatically extracted, as also posed by the ANS theory (Content et al., 2017; de Hevia et al., 2017; Libertus et al., 2017; Nieder, 2017; Park et al., 2017; Savelkouls and Cordes, 2017; Stoianov and Zorzi, 2017).

In the current study, we aimed to give further insight into the processing of non-symbolic numerosities. Therefore, we examined the timing of ratio-related distance effects in the ERP while using larger quantities and stringent control over visual properties by using the method of Gebuis and Reynvoet (2011). Based on the ANS theory, one would expect early ratio-related distance effects in the ERP, suggesting processing of numerosity independent of visual properties. However, an absence of early effects for numerosity in combination with longer lasting effects based on visual properties, would suggest that visual properties of stimuli are not removed to approximate numerosity, but visual properties do play a role in determining numerosity. An absence of the ratio-related distance effect would support the previous findings discussed above (Gebuis and Reynvoet, 2012, 2013; Soltész and Szűcs, 2014). However, these studies examined passive viewing of dot patterns (Gebuis and Reynvoet, 2012, 2013; Soltész and Szűcs, 2014), in which the attention of the participants was not directed toward the numerosity of the set. Only in the second experiment in the study of Gebuis and Reynvoet (2013), participants were instructed to attend to the numerosity by including attention trials on which the participant needed to estimate the numerosity of the current stimulus. However, manipulation of the distance or ratio between two stimuli, as more generally used in ERP and behavioral research on numerosity processing (Moyer and Landauer, 1967; Temple and Posner, 1998; Libertus et al., 2007; Paulsen and Neville, 2008; Hyde and Spelke, 2009) is lacking.

### The ANS Mapping Account

The ANS is not only the most prominent theory on non-symbolic number processing, but also the basis for the most common model for the processing of symbolic numbers. This model on symbolic number processing based on the ANS is referred to as the ANS mapping account. The core of the ANS mapping account is that adults intuitively map symbolic numbers onto the corresponding non-symbolic numerosity before further processing (Dehaene, 1997). As such, a comparison task with symbolic stimuli is solved in a manner similar to a non-symbolic comparison task after mapping the symbolic number onto the non-symbolic numerosity.

The ANS mapping account is supported by symbolic comparison tasks that show effects similar to the ratio effect found for non-symbolic stimuli. More specifically, behavioral performance on symbolic comparison tasks reflects distance and size effects (Dehaene et al., 1990; Verguts and Van Opstal, 2005; Holloway and Ansari, 2008; Sasanguie et al., 2012, 2013). The distance effect entails better performance when two quantities are further apart from each other, whereas the size effect entails better performance for small numerosities as compared to large numerosities when the distance between them is equal (i.e., 3 vs. 4 is easier to compare than 7 vs. 8). Together, the distance and size effects are similar to the ratio effect found in non-symbolic comparison tasks (Holloway and Ansari, 2008; Halberda et al., 2012; Sasanguie et al., 2012, 2013), which is thought to support the ANS mapping account (see Reynvoet and Sasanguie, 2016 for a review). ERP studies have shown that the timing of these effects is also similar to the ratio-effects found in non-symbolic processing (Dehaene, 1996; Temple and Posner, 1998; Libertus et al., 2007). Together, these results suggest that the processing of symbolic number relies on the processing of non-symbolic numerosity.

However, the underlying assumption that distance effects found in behavioral and ERP research reflect overlapping neural representations has been questioned. Research has shown that the distance effect found in comparison tasks, hence called the comparison distance effect (CDE), does not necessarily originate from the larger overlap in neural representation in two numerically close numbers, but may be caused by more general decision processes (Van Opstal et al., 2008). Comparison tasks with letters and digits were compared to each other. Participants had to indicate whether a digit between 1 and 9 was smaller or larger than 5, and whether a letter between J and R came either before or after the letter N in the alphabet. A CDE was found for both letters and digits, even though letters are not assumed to have overlapping neuronal representations with neighboring letters, suggesting that the distance effects found in comparison tasks do not necessarily support the ANS mapping account.

In the same paper, Van Opstal et al. (2008) re-analyzed the data from the comparison task based on the distance between the previous digit or letter (the prime) and the current number or letter (the target). They showed that reaction times were shorter when the digit in the previous trial was close to the digit presented in the current trial (4 preceded by 3) than when the digit in the previous trial was further away from the one presented in the current trial (4 preceded by 1). This faster reaction is assumed to be due to the fact that the quantity was already partly activated, and thus primed, during processing of the previous digit, and hence named the prime distance effect (PDE). This effect was found to be specific for digits, and not present for letters. In a follow-up study, van Opstal and Verguts (2011) found that the PDE was not limited to the specific task described above, but could also be found in a same-different match-to-sample task. In this task, participants were presented with two symbolic numbers (a digit and a number word) consecutively and had to respond to indicate whether these stimuli depict the same or a different quantity. For stimuli that differed from each other, the distance between the prime (number that is presented first) and the target (number that is presented second) was manipulated. Reaction times to the “different” targets were faster when the numbers were further apart from each other (e.g., 2 vs. 8) than when the numbers were close to each other (e.g., 7 vs. 8). This was interpreted as an effect of more co-activation due to overlapping neural representations in the latter case. However, this study did not examine whether these distance effects for symbolic numbers were related to distance effects found for non-symbolic stimuli. Behavioral evidence shows low correlations between the distance effects in symbolic and non-symbolic tasks, questioning whether these tasks are solved based on similar processing in both tasks (Holloway and Ansari, 2009). Also, recent research shows that although the ANS model can describe behavioral results in non-symbolic tasks relatively well, it has difficulty in describing behavioral results in symbolic comparison tasks, again indicating that symbolic numbers are not processed by the ANS (Krajcsi et al., 2018).

To directly investigate similarities between symbolic and non-symbolic processing, mapping tasks in which symbolic and non-symbolic quantities need to be compared to each other should be used. Based on the ANS mapping account that symbolic processing is rooted in non-symbolic numerosity processing, one would expect that results in purely symbolic tasks, purely non-symbolic tasks, and tasks in which symbolic and non-symbolic numbers need to be combined are similar. More specifically, one would expect that the processing of non-symbolic numerosities would not be affected by the format of the stimulus it needs to be compared to. As such, based on the ANS mapping account, one would not expect differences between the primes in the purely non-symbolic task and the mapping task with non-symbolic primes and symbolic targets. Similarly, one would not expect differences between the processing of non-symbolic targets in the purely non-symbolic task and the mapping task with symbolic primes and non-symbolic targets. Behavioral evidence from mapping tasks shows that performance on mapping tasks is worse than performance on a purely non-symbolic comparison task (Lyons et al., 2012), suggesting that the mapping of symbolic numbers onto non-symbolic numerosities is not an intuitive process. Another study showed that tasks involving non-symbolic stimuli elicit a ratio effect, both completely non-symbolic tasks as well as when mapping tasks. However, purely symbolic tasks did not show a ratio effect. This suggests that the non-symbolic numerosity in the ANS may not be activated when comparing two symbolic numbers (Sasanguie et al., 2017).

These data question the validity of the ANS mapping account in two ways. First of all, they question whether symbolic numbers are mapped onto non-symbolic numerosities when this is not necessary for the task at hand. Second, they question whether the possible mapping occurs intuitively. Therefore, in the current study, we measured ERPs in same-different match-to-sample tasks with symbolic stimuli, non-symbolic stimuli, or a combination between symbolic and non-symbolic stimuli to examine whether symbolic numbers are indeed mapped onto non-symbolic numerosities, and if so, whether this mapping is an automatic process. The ANS mapping account is examined in two ways. First, based on the ANS mapping account, one would expect similar distance effects in symbolic and mapping tasks as in the non-symbolic task if symbolic numbers are indeed mapped onto the ANS. Second, one would expect that the non-symbolic stimuli are processed similarly resulting in similar ERPs, regardless of whether they need to be compared to symbolic stimuli or non-symbolic stimuli, since the ANS is the core system, which is at the basis of numerical processing. Stated otherwise, a difference in the ERPs for non-symbolic stimuli depending on the task suggests that this does not lie at the basis of numerical processing. This would provide evidence against the ANS mapping account.

## Materials and Methods

### Participants

Twenty-three adults, mainly undergraduate students, participated in the study. Two were excluded due to noisy EEG data (see below). The final sample consisted of four males and 17 females, with a mean age of 23 years and 10 months (SD 3 years, 3 months). Of the participants, 19 were right handed, and 2 were left handed. All participants had normal or corrected-to-normal vision. All participants gave written informed consent in accordance with the Declaration of Helsinki.

### Procedure

Participants were seated in an electrically shielded room. They were informed that the study would assess numerical skills and consisted of four comparison tasks. Upon successful application of the EEG, the task instruction of the first task was presented on the screen. Participants were told that there would be a break after each task. During these breaks the researcher would come in to ask how they were doing and to answer any questions. The tasks were presented in a fixed order with the non-symbolic task first, then the non-symbolic/symbolic task, then the symbolic/non-symbolic task, and finally the symbolic task. The order of the tasks was fixed such that participants did not know which numbers were presented in the non-symbolic format and were not able to calculate the ratios based on the purely symbolic task. After the four tasks, the EEG cap was removed from the participant and they were financially compensated for participation with 10 Euros. All tasks including application and removal of the EEG-cap lasted about 75 min.

### Tasks

#### Non-symbolic (Ns-Ns)

**FIGURE 1:**
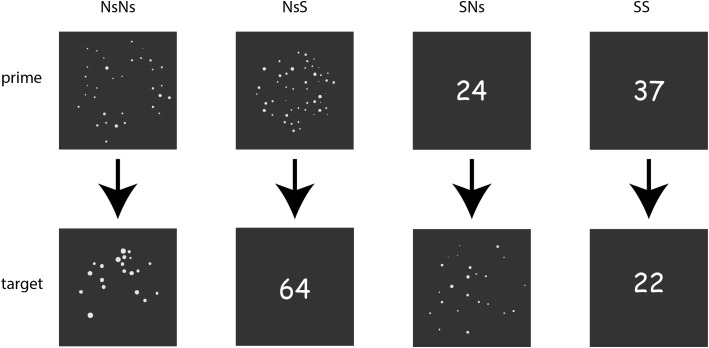
Overview of the stimuli-formats in the different tasks with the upper line presenting the primes for each task, and the lower line presenting the targets.

In the non-symbolic task, trials consisted of a prime picture with a dot pattern and a target picture with a dot pattern, see **Figure 1**. The dot patterns were generated in MATLAB with the script described in Gebuis and Reynvoet (2011). Using this script, the relation between the number distance and visual properties was controlled, as well as the congruency in area subtended, density, total surface of the dots, average diameter, and total circumference. Moreover, visual properties of the stimuli are documented, which gives the opportunity to divide data based on visual properties as well (Gebuis and Reynvoet, 2011). The number of dots for the primes ranged between 20 and 40, with both smaller and larger targets at ratio 0.5, 0.6, and 0.7. As such, all numbers ranged between 10 and 80, and thus far out of the subitizing range. A trials started with the presentation of a prime for 750 ms, then a blank screen jittered between 400 and 600 ms, and a target presented for 750 ms. The inter trial interval was jittered between 1,000 and 1,500 ms. Thirty trials were presented for each distance x size (target larger vs. target smaller than prime). In 10 percent of the trials (20 trials), the numerosity in the prime and the target were the same, resulting in a total of 200 trials^1^. Participants were instructed to passively watch the stimuli and only respond by pressing the space bar if they thought the prime and target stimuli displayed the same quantity.

#### Non-symbolic – Symbolic (Ns-S)

The Ns-S task was identical to the Ns-Ns task with the exception that the targets were presented as digits instead of dot patterns.

#### Symbolic – Non-symbolic (S-Ns)

The S-Ns task was identical to the Ns-Ns task with the exception that the primes were presented as digits instead of dot patterns.

#### Symbolic (S-S)

The S-S task differed slightly from the Ns-Ns task. Both the prime and the target were presented as digits. Moreover, the stimuli were presented for 500 ms instead of 750 ms, since the task was very simple.

### Analyses

#### Behavioral

Participants had to respond only to trials in which they thought the prime and target matched each other. As such, a non-response to the trials in which the prime and target did not match each other is taken as a correct response. Behavioral data were analyzed in SPSS, version 23. Proportions correct were analyzed per task in a Ratio (0.5, 0.6, and 0.7) ^∗^ Size (target larger vs. target smaller) repeated measures ANOVA. Polynomial contrasts were included to test whether performance increased linearly with decreasing ratio.

### ERP

#### Recording and Preprocessing

Data were recorded with a 32 electrode active cap (Biosemi, Amsterdam, Netherlands) with a sampling rate of 2048 Hz. The electrode offset was kept below 50 μV. Data were recorded without reference. After recording, data were imported into MATLAB 2015a (The MathWorks Inc., Natick, MA, United States) and analyzed using the Fieldtrip toolbox (Oostenveld et al., 2011).

Data were downsampled to 512 Hz, rereferenced to the linked mastoids, and low-pass filtered at 40 Hz. ICA was used to identify and delete eye blinks and horizontal eye movements. After that, data were manually inspected for bad channels. Bad channels were removed and replaced with a weighted sum of the surrounding channels. Removed channels were never adjacent to each other. Data (primes and targets) were segmented from 200 ms before to 750 ms after stimulus onset and baseline corrected. After artifact rejection, the data were averaged per ratio per task for the targets and averaged per task for the primes. Data from target larger than prime and target smaller than prime were collapsed because of the limited number of trials included. Next to that, averages were generated for small, medium, and large diameter; small, medium, and large area; and small, medium, and large surface. The averages were created such that they contained the same number of trials as the averages per ratio.

#### Analyses

Single-subject averages were included in the analyses if at least 40 artifact free trials were included in the average for each condition. Since the time course of the differences between conditions was unknown, cluster based permutation tests were carried out. For the Ratio effects in the tasks, four separate permutation tests were carried out, one for each task. A linear effect of Ratio was expected. Therefore, the t-statistic of the slope of a multilevel linear estimation procedure with fixed slope and random intercept was used as input for the analyses. Similar cluster based analyses were performed for the physical parameters (mean) diameter, area (within the convex hull), and total surface (of the dots).

To test for differences in the processing of non-symbolic stimuli depending on task, two cluster based permutation tests were carried out, one to compare the processing of primes in the NsNs-task vs. the NsS-task, and one to compare the processing of the targets in the NsNs-task vs. the SNs-task. A dependent-samples *t*-test was used as input for the cluster based permutation test. Since cluster-based statistics (clusterstats) are calculated for positive and negative clusters separately, the *p*-values will be compared to α = 0.025 (0.05/2) for all analyses.

## Results

### Behavioral

**FIGURE 2:**
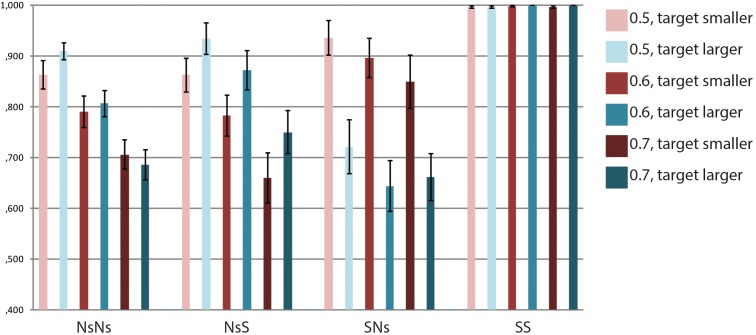
Accuracy data per Ratio × Size for each of the tasks.

Accuracy data for each task are presented in **Figure 2**. For the NsNs-task, the repeated measures ANOVA with the factors Ratio (0.5, 0.6, and 0.7) and Size (target smaller and target larger) revealed a main effect of Ratio, *F*(2,40) = 53.03, *p* < 0.001, but no significant effect of Size, *F*(1,20) = 0.36, *p* = 0.554, and no interaction between Ratio and Size, *F*(2,40) = 1.89, *p* = 0.165. The polynomial contrasts showed a linear trend for Ratio, *F*(1,20) = 75.12, *p* < 0.001, but no quadratic trend, *F*(1,20) = 0.34, *p* = 0.569. For the NsS-task the results were similar. A main effect of Ratio was found, *F*(2,40) = 33.54, *p* < 0.001, but no effect of Size, *F*(1,20) = 1.86, *p* = 0.188, and no interaction between Ratio and Size, *F*(2,40) = 0.14, *p* = 0.803. The polynomial contrasts indicated a linear trend as well as a quadratic trend, *F*(1,20) = 38.71, *p* < 0.001 and *F*(1,20) = 4.92, *p* = 0.038, respectively. This indicates that accuracy increases with the smaller ratio’s, and the difference in accuracy is larger between 0.6 and 0.7 than between 0.5 and 0.6. For the SNs-task, a main effect of Ratio, *F*(2,40) = 4.00, *p* = 0.049, and a main effect of Size were found, *F*(1,20) = 8.30, *p* = 0.009. No interaction between Ratio and Size, *F*(2,40) = 0.95, *p* = 0.361 was present. The results show higher accuracy when the target was smaller than the prime as compared to when the target was larger than the prime. The polynomial contrasts indicated marginally significant linear and marginally significant quadratic trends, *F*(1,20) = 4.03, *p* = 0.058 and *F*(1,20) = 3.76, *p* = 0.067. In the SS-task, no significant main effects of Ratio and Size, *F*(2,40) = 0.77, *p* = 0.470 and *F*(1,20) = 1.88, *p* = 0.186, respectively, and no interaction between Ratio and Size were found, *F*(2,40) = 0.59, *p* = 0.560.

#### Ratio Effects Targets

**FIGURE 3:**
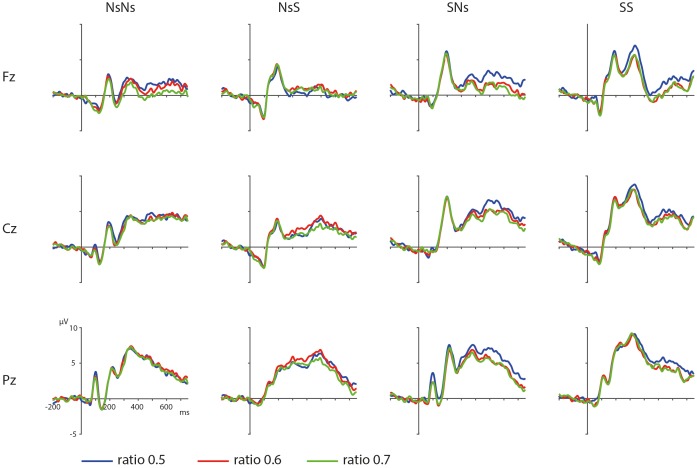
Distance effects on frontal, central, and parietal electrodes in the different tasks with the blue waveform depicting targets with a ratio 0.5, red depicting a ratio of 0.6, and green depicting a ratio of 0.7.

ERPs depicting the ratio effects of the targets in the different tasks are shown in **Figure 3**. The results of the permutation test on the ratio effect in the Ns-Ns task shows no significant cluster for ratio, largest positive clusterstat = 1376.4, *p* = 0.846, and largest negative clusterstat = -4899.1, *p* = 0.094. For the NsS task, no significant cluster for ratio was found either, largest positive clusterstat = 2529.4, *p* = 0.902, largest negative clusterstat = -4985.4, *p* = 0.246. For the SNs task, no significant cluster for Ratio was found, largest positive clusterstat = 2459.7, *p* = 0.816, largest negative clusterstat = -2967.3, *p* = 0.339. For the SS task, results showed no significant clusters either, largest positive clusterstat = 2779.7, *p* = 0.994, largest negative clusterstat = -587.4, *p* = 0.118. These results reflect an absence of an effect for Ratio for all tasks.

#### Differences Between Tasks

Since no ratio effects were found, the timing of the ratio effects in the different tasks could not be compared. Hence, differences between tasks were only assessed based on the differences in the processing of the non-symbolic stimuli.

**FIGURE 4:**
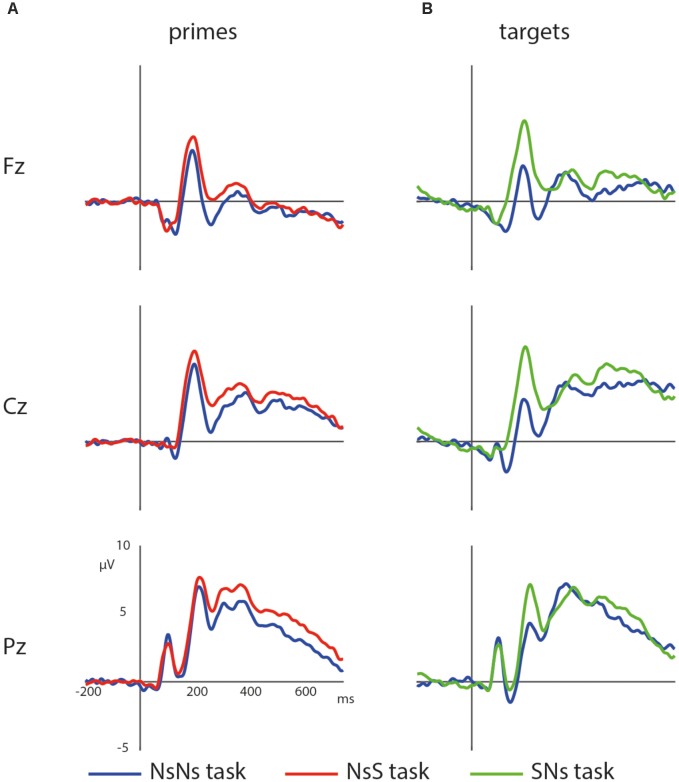
Waveforms of the non-symbolic primes **(A)** and non-symbolic targets **(B)** in the different tasks with the NsNs-task in blue, the NsS-task in red, and the SNs-task in green.

**FIGURE 5:**
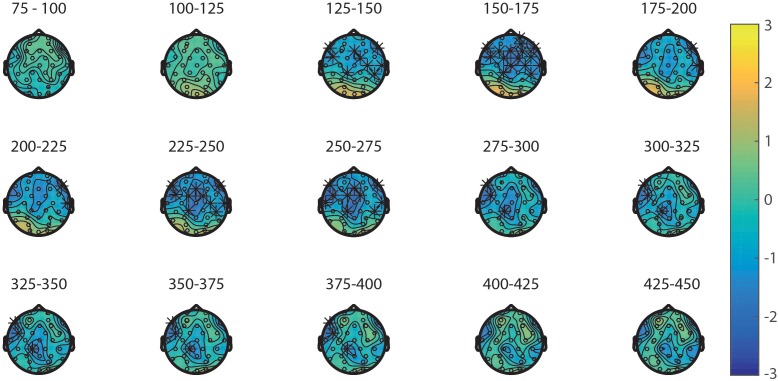
Topoplots of the differences between the primes in the NsNs-task and NsS-task per time window with stars representing the significant differences between the tasks.

First, the processing of the primes in the NsNs-task and the NsS-task was compared. ERPs of the primes in these tasks are depicted in **Figure 4A**. The results of the permutation test on the primes in the NsNs and NsS task revealed a significant negative cluster, clusterstat = -3777.7, *p* = 0.022, but no significant positive cluster, largest clusterstat = 627.3, *p* = 0.060. The negative cluster reflects a fronto-central negativity between 125 and 400 ms, being relatively widespread between 125 and 175 ms, moving to mainly left-frontal between 275 and 400 ms (see **Figure 5**).

**FIGURE 6:**
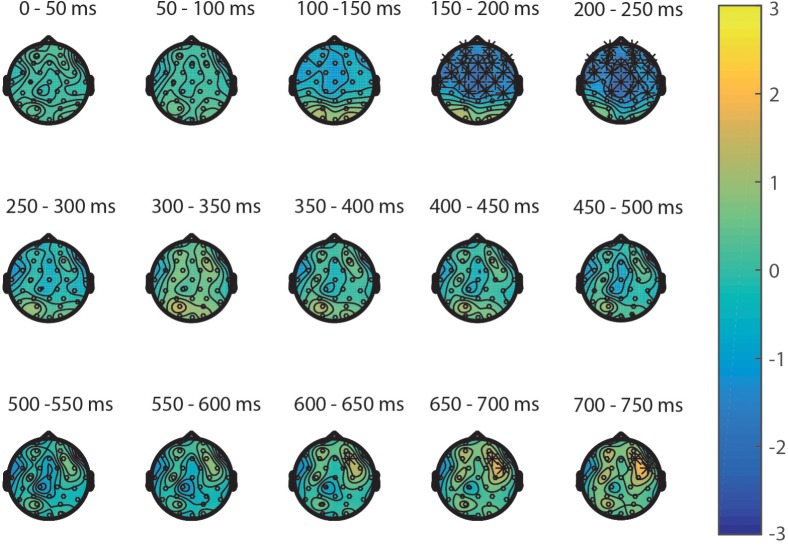
Topoplots of the differences between the targets in the NsNs-task and SNs-task per time window with stars representing the significant differences between the tasks.

Second, the processing of targets in the NsNs-task was compared to the processing of the targets in the SNs-task. ERPs depicting the processing in both tasks are shown in **Figure 4B**. The permutation test on the difference between non-symbolic targets in the NsNs task and SNs task shows a significant positive cluster, clusterstat = 4243.8, *p* = 0.012 reflecting a right-frontal difference between 600 and 750 ms, and a significant negative cluster, clusterstat = -7613.6, *p* = 0.002 reflecting a widespread fronto-central negativity between 150 and 250 ms (see **Figure 6**).

#### Visual Properties of Non-symbolic Stimuli

**FIGURE 7:**
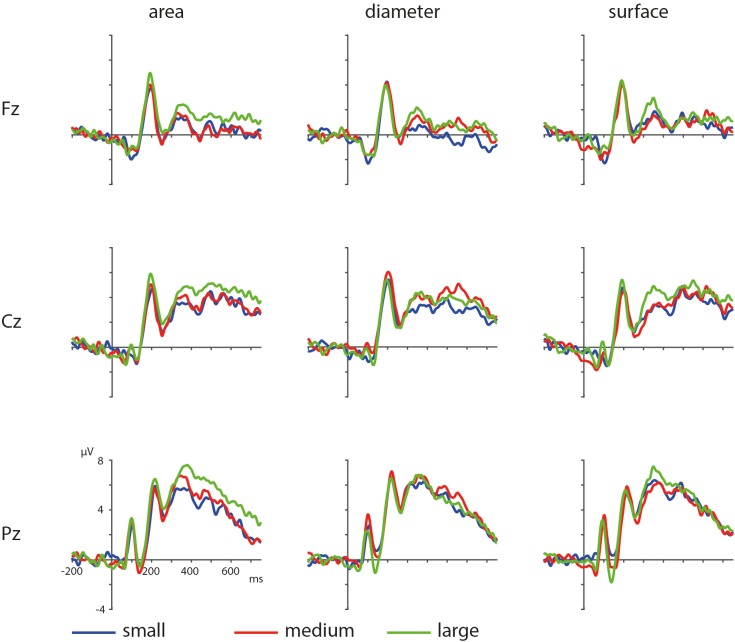
ERPs based on visual parameters area, diameter and surface with blue depicting small, red depicting medium, and green depicting large area/diameter/surface.

**FIGURE 8:**
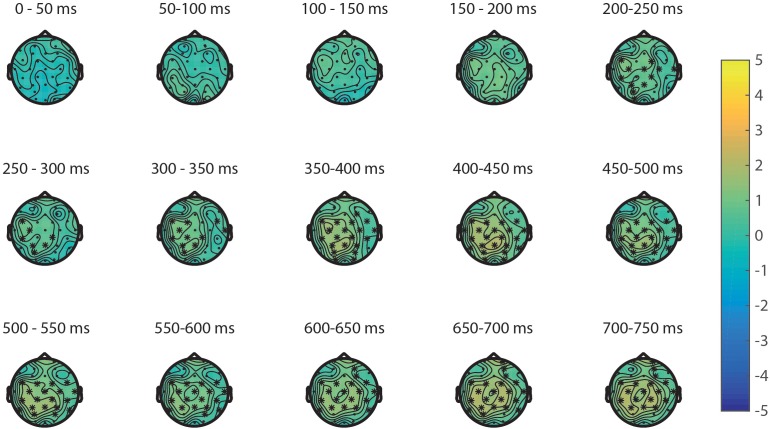
Significant cluster for area plotted on the topoplots of the differences between small area and large area.

The ERPs of the visual properties are displayed in **Figure 7**. With regards to the visual properties, the results of the permutation test on area showed a positive cluster, clusterstat = 24642, *p* = 0.008, but no significant negative cluster, largest clusterstat = -2583.8, *p* = 0.571. This cluster reflects a widespread positivity increasing with area covered between 200 and 750 ms in fronto-central to parietal regions (see **Figure 8**). The results of the permutation test on diameter showed no significant positive and negative cluster, with the largest clusters being, respectively, clusterstat = 17500, *p* = 0.108 and clusterstat = -2241.4, *p* = 0.465. The results of the permutation test on surface show no significant positive cluster, largest clusterstat = 2298.4, *p* = 0.082, and no significant positive cluster, largest clusterstat = -4005.7, *p* = 0.353.

## Discussion

The ANS theory and ANS mapping account (Dehaene, 1997) have been the most prominent theories on number processing in the past decades. However, recently, the validity of the ANS theory and ANS mapping account have been questioned. The aim of the current study was twofold. First, we examined whether non-symbolic numerosity is processed intuitively and independent of the processing of visual features as claimed by the ANS theory. Next, we examined whether symbolic numbers are mapped onto non-symbolic numerosities, as expected based on the ANS mapping account. ERPs were measured during a same-different match-to-sample task with non-symbolic numerosities, a task with symbolic numbers, and mapping tasks in which the prime was symbolic and the target non-symbolic or vice versa.

### ANS Theory

As support for the ANS theory, one would expect (early) distance effects in the completely non-symbolic task. Our results show that despite the distance effect in the behavioral data, no ERP distance effects for numerosity were found, which means that the ratio between the numerosity of the prime and target was not visible in the ERP signal. This result is in line with previous research using strict control over visual properties (Gebuis and Reynvoet, 2012), and suggests that numerosity is not intuitively activated in non-symbolic stimuli. In contrast, the ERP results do show an early distance effect starting at 200 ms when stimuli are categorized based on the visual property area instead of numerosity, indicating that area is processed very quickly. This suggests that the area subtended by the convex hull around the dots is activated and processed. These results are in contrast with previous research in which processing of numerosity was claimed based on numerosity-related distance effects with non-symbolic stimuli (Temple and Posner, 1998; Libertus et al., 2007; Paulsen and Neville, 2008; Hyde and Spelke, 2009, 2012). In those studies, visual properties were not controlled for in a strict manner, resulting in the possibility to use visual properties to inform oneself about numerosity. In studies with proper control, Gebuis and Reynvoet (2012, 2013) also found effects for visual processing, but not for numerosity processing. This confirms that the early effects found in the abovementioned studies are likely due to insufficient control over visual properties, as suggested by Gebuis and Reynvoet.

An alternative explanation for the lack of a distance effect for numerosity is that the ANS theory does hold, but that this distance effect cannot be measured with ERP. Most models on the ANS theory suggest that individual objects go through a normalization phase in which sensory properties are removed before they enter the accumulator stage in which the information is transformed into numerosity (Dehaene and Changeux, 1993). Whereas a lack of a distance effect for numerosity in the ERP does not necessarily contradict to this idea, the presence of a long lasting distance effect for area, up until 750 ms, does. If the stimuli would go through a normalization phase, one would expect only effects of visual properties before this stage, i.e., only very early in the ERP. Thus, our data support the claim that a normalization phase is unlikely (Gebuis et al., 2016). Taken together, our ERP results do not support the ANS theory. However, the behavioral distance effect suggests that approximate numerosity is established. Our ERP results suggest that this is achieved based on the processing of the visual properties. This is in line with previous research showing that visual properties are processed more automatically as compared to numerosity (Gebuis and Reynvoet, 2013; Smets et al., 2015). As alternatives for the ANS theory, the sensory integration theory and sense of magnitude theory have been proposed (Gebuis et al., 2016; Leibovich et al., 2017). Our results with large and long-lasting distance effects for area and not numerosity, support these theories by showing that magnitude (in this case area) is processed more automatically than numerosity.

### ANS Mapping Account

The second aim of our study was to examine the ANS mapping account. Whereas the results of the non-symbolic task question the existence of the ANS theory in its current form, mapping of symbolic stimuli onto their non-symbolic counterparts may still occur. The first line of evidence for the ANS mapping account would come from similar distance effects in the non-symbolic task and the symbolic and mapping tasks. The behavioral results shows similar distance effects in the non-symbolic and mixed tasks, but no distance effect in the purely symbolic task, which is in line with recent research (Sasanguie et al., 2017). This strengthens the claim that indeed in purely symbolic tasks, non-symbolic numerosity is not activated. The ERPs showed no distance effect in any of the tasks. Due to the lack of distance effects in the ERP, comparing these ERP distance effects between tasks is not possible.

The second line of evidence for the ANS mapping account would come from similar processing of non-symbolic stimuli regardless of task. Distance effects are no prerequisite to examine these similarities or differences. If symbolic number processing is rooted in non-symbolic numerosity processing, then the processing of the non-symbolic stimulus should not be affected by the format of the stimulus to which it needs to be compared. Whereas similar behavioral distance effects were found for all tasks including non-symbolic stimuli, our ERP results show differences in the processing of the primes between the purely non-symbolic task in which two dot patterns were presented and the mapping task with non-symbolic primes (dot patterns) and symbolic targets (digits). Moreover, differences between the targets in the purely non-symbolic task and the mapping task with symbolic primes and non-symbolic targets were found. Processing of non-symbolic numerosity is thus affected by task, which is highly unlikely in the light of the ANS mapping account. However, the results could possibly still support the account, if the ERPs in the mapping tasks would show highly similar, but slightly delayed waveforms in the mapping task as compared to the non-symbolic task. Visual inspection of the waveforms does not support this. Instead, differences seem to occur mainly in amplitude instead of latency. For the non-symbolic primes, the amplitude in the mapping task was more positive between 125 and 400 ms than in the purely non-symbolic task on the anterior electrodes. For the targets, the amplitude was more positive for the mapping task as compared to the purely non-symbolic task between 115 and 275 ms and more positive for the purely non-symbolic task than the mapping task between 578 and 750 ms. These differences both early and late in the processing stream suggest that different cognitive processes take place in the different tasks. As such, the data do not support the ANS mapping account. It suggests that symbolic stimuli are not intuitively mapped onto their non-symbolic counterparts, even when the task requires mapping. This is in line with previous research on mapping (Lyons et al., 2012) and studies showing a lack of correlation between distance effects in non-symbolic and symbolic tasks (Holloway and Ansari, 2009; Sasanguie et al., 2017).

A recent alternative to the ANS mapping account is symbolic processing based on symbol–symbol associations (Reynvoet and Sasanguie, 2016). This account suggests that whereas small symbolic numbers initially acquire meaning through mapping, larger symbolic numbers are learned through associations between symbolic numbers, such as “order” and “the successor function” (Carey, 2001, 2004, 2009). In adulthood, symbolic and non-symbolic numerosities would be processed independent from each other if tasks do not require relating them to each other (Lyons et al., 2012; Sasanguie et al., 2017). Both our behavioral and ERP data support this idea, as shown by the differences in ERPs between the tasks. However, the account on symbol–symbol associations does not directly lead to any predictions for mapping tasks.

In mapping tasks, contrary to what was proposed in the ANS mapping account, it may be the case that non-symbolic numerosities are first estimated and then compared to the symbolic number based on the symbol–symbol account. This may also explain the differences between the processing of the non-symbolic stimuli in the non-symbolic task vs. the mapping tasks. If a non-symbolic numerosity needs to be compared to a symbolic number, then it may first need to be estimated. However, if a non-symbolic numerosity needs to be compared to another non-symbolic numerosity, this is not necessary, which is in line with the differences we found in the ERPs. This is also supported by research showing longer reaction times in mapping tasks involving symbolic and non-symbolic stimuli (Lyons et al., 2012). Additional support for this claim would come from similar processing of symbolic stimuli in the symbolic and mapping tasks. However, our paradigm does not allow to test this hypothesis, since the symbolic task did not require participants to process quantity at all. Since the same format (digits) was used for the primes and the target, the task could be performed by visual matching instead of matching based on quantity. Therefore, neither the ERPs nor the behavioral data give insight into the processing of symbolic number. Future research should include a different symbolic task, for example with number words and digits, to make sure participants process the numerical magnitude of the stimulus.

Taken together, our results support the converging evidence against the ANS theory and the ANS mapping account (Gebuis et al., 2016; Reynvoet and Sasanguie, 2016; Leibovich et al., 2017; Núñez, 2017). However, our lack of distance effects was based on null results. Whereas the analyses on the visual features with the same power did produce statistically significant results, the conclusions need to be interpreted with some caution. Research with a different paradigm showing similar results would strengthen our conclusions. For now, the results in the non-symbolic task do support the sensory-integration theory for processing non-symbolic numerosity (Gebuis et al., 2016) or sense for magnitude theory (Leibovich et al., 2017) instead. We suggest that mapping may be a two-step process, consisting of dot enumeration followed by comparison based on symbol–symbol associations (Reynvoet and Sasanguie, 2016). Future research including mapping tasks with purely symbolic stimuli, such as number words and Arabic numbers may shed further light on this issue.

## Ethics Statement

This study was carried out in accordance with the recommendations of the ethics committee of the Faculty of Social and Behavioral Sciences of the University of Utrecht. The protocol was approved by the ethics committee of the Faculty of Social and Behavioral Sciences of the University of Utrecht. All subjects gave written informed consent in accordance with the Declaration of Helsinki.

## Author Contributions

All authors listed have made substantial, direct, and intellectual contributions to the work, and approved for publication. AvH and EK construed the study together. AvH gathered the data together with research assistants and master students. Analyses were carried out by AvH. AvH and EK wrote the paper together.

## Conflict of Interest Statement

The authors declare that the research was conducted in the absence of any commercial or financial relationships that could be construed as a potential conflict of interest.

## Footnotes

1 Stimulus generation failed for 18 out of 400 stimuli. As such, instead of 200 trials, 184 trials were presented in the Ns-Ns task, 187 trials in the Ns-S task, and 195 trials in the S-Ns task.
